# The Evolutionary Consequences of Disrupted Male Mating Signals: An Agent-Based Modelling Exploration of Endocrine Disrupting Chemicals in the Guppy

**DOI:** 10.1371/journal.pone.0103100

**Published:** 2014-07-21

**Authors:** Alistair McNair Senior, Shinichi Nakagawa, Volker Grimm

**Affiliations:** 1 Zoology Department, The University of Otago, Dunedin, Otago, New Zealand; 2 The Charles Perkins Centre, The University of Sydney, Sydney, New South Wales, Australia; 3 School of Biological Sciences, The University of Sydney, Sydney, New South Wales, Australia; 4 Department of Ecological Modelling, Helmholtz Centre for Environmental Research, Leipzig, Saxony, Germany; 5 Institute for Biochemistry and Biology, University of Potsdam, Potsdam, Brandenburg, Germany; University of Missouri, United States of America

## Abstract

Females may select a mate based on signalling traits that are believed to accurately correlate with heritable aspects of male quality. Anthropogenic actions, in particular chemicals released into the environment, are now disrupting the accuracy of mating signals to convey information about male quality. The long-term prediction for disrupted mating signals is most commonly loss of female preference. Yet, this prediction has rarely been tested using quantitative models. We use agent-based models to explore the effects of rapid disruption of mating signals. In our model, a gene determines survival. Males signal their level of genetic quality via a signal trait, which females use to select a mate. We allowed this system of sexual selection to become established, before introducing a disruption between the male signal trait and quality, which was similar in nature to that induced by exogenous chemicals. Finally, we assessed the capacity of the system to recover from this disruption. We found that within a relatively short time frame, disruption of mating signals led to a lasting loss of female preference. Decreases in mean viability at the population-level were also observed, because sexual-selection acting against newly arising deleterious mutations was relaxed. The ability of the population to recover from disrupted mating signals was strongly influenced by the mechanisms that promoted or maintained genetic diversity in traits under sexual selection. Our simple model demonstrates that environmental perturbations to the accuracy of male mating signals can result in a long-term loss of female preference for those signals within a few generations. What is more, the loss of this preference can have knock-on consequences for mean population fitness.

## Introduction

Inter-sexual selection, resulting from heritable preferences for mates bearing specific traits, strongly influences the evolutionary trajectory of many species [1]. Often, females select a mate from the many available using signal traits such as large size or elaborate ornaments, which correlate with breeding value or ‘quality’. In doing so, females may obtain direct and/or indirect benefits [1], [2]. For example, under a good-genes scenario, signal traits may correlate with genetic factors that increase survival [3]. Hence, females choosing to mate with a male that bears such a trait benefit indirectly by selecting a father that can contribute genes that enhance offspring viability. Consequently, females showing preference for this signalling trait will, on average, have higher-quality offspring than females that show no preference, and will simultaneously pass on their preference for that trait.

Systems of mate-choice that confer indirect genetic benefits evolve and are maintained because the relationship between signal and breeding value is accurate; perhaps because the signal is too costly to ‘fake’ or because all males invest in signalling traits relative to their quality [4]. However, the ability of females to perceive, and of males to express, signals accurately also relies on the environment [5]–[7]. Anthropogenic actions are now changing environments at a rate that far surpasses any previously encountered in the evolutionary history of most taxa [8]. In some instances, environmental change, such as the presence of exogenous chemicals or urban noise, may disrupt the relationship between signals of male ‘mating value’ and the ‘true’ breeding value of a male [9]–[11]. Human-induced environmental changes have therefore been predicted to have consequences for systems of inter-sexual selection [10], [12]–[15]. At the population level the most common prediction is loss of female preference, because the benefits of expressing mating preferences are negated when signals are unreliable [10], [16], [17]. However, the consequences of disrupted mating signals may be as serious as reductions in population viability depending on the nature of the disruption and the system in question [10], [12], [15]–[18].

For example, disrupted mating signals have been observed in one of the most renowned examples of inter-sexual selection, the guppy (*Poecilia reticulata*). Female guppies display preference for males that bear orange spots [19]–[21]. These markings correlate with traits that are essential to survival, such as foraging ability and immunocompetence, and hence are thought to indicate male genetic quality and viability [21]–[25]. The area of orange spots on male guppies can be reduced by pollutants known as endocrine-disrupting chemicals (EDCs) [26], [27]. EDCs are exogenous chemicals, which disrupt hormonal regulation in exposed individuals [28], [29], and have been suggested to affect intersexual selection in a range of taxa, as well as guppies [10], [15].

Understanding the short- and long-term effects of disrupted male sexual signalling will be achieved through multi-generational experimental studies. However, these studies require significant investment, which may not be justifiable without clear predictions. Thus far, predictions of the effects of environmental perturbations (such as EDCs) on systems of inter-sexual selection have rarely gone further than verbal arguments. Agent-based models (ABMs) can be used to simulate sexual selection [30], allowing preliminary long-term predictions to be obtained in a relatively short time [31]. Here, we present the results of an ABM designed to explore the effects of disrupted male mating signals in a simple system of inter-sexual selection, with stochasticity and overlapping generations.

Within our model, a quantitative heritable trait confers a survival, or viability, advantage (e.g. genes that improve foraging ability or immunocompetence in guppies), and the values of this trait vary among individuals. Males signal their mean allelic value for this trait (e.g. orange spots in guppies). For simplicity, we assume that males honestly and directly signal their genetic quality. However, the expression of signal may be disrupted by an environmental parameter (e.g. EDCs). Females express a heritable preference for male signals (e.g. preference for orange spots in guppies). We parameterise the model to maximise the correlation between evolved signal and female preference in the absence of disrupted mating signals, thus representing inter-sexual selection. We allow this system to evolve before introducing disruption of the relationship between male signal and survival for a period. Finally, we allow the environment to return to its original state. In this way, the model will provide mechanistic insights in to the potential breakdown and recovery of sexual selection.

Our primary aim was to test predictions that female preference will be lost in the face of rapid environmental change that disrupts male-quality signals. We estimate the number of generations over which any preference loss occurs and whether the evolutionary consequences of disrupted mating signals endure once the environment is returned to its original state. Our model was inspired by the guppy system outlined above and therefore takes life-history parameter values (e.g. fecundity and longevity) representing guppies. However, our overall approach is not specific to endocrine-disruption of mating signals in this species. Our model is designed as an initial exploration of the effects of disrupted sexual selection in a simplified system; in particular, our representation of genes, their expression, and selection acting upon them is simplistic. This approach allows us to produce preliminary generalisable results, whilst keeping the contribution of model components those results tractable.

## Methods

### The Model

The model description follows the ODD (Overview, Design concepts, Details) protocol for describing agent-based models [32], [33]. The model was implemented in NetLogo, a free software platform for implementing agent-based models [34]. NetLogo code for this model can be found in File S2: Model Code.

#### Purpose

This model examines the effects of rapid environmental perturbations of male mating signals on systems of inter-sexual selection.

#### Entities, States, Variables and Scales

The model includes three types of individuals, males, females and juveniles, and the environment. Individuals have the following state variables: *Age* (in time steps), and the traits *Viability, Signal* and *Preference* (all numeric variables). *Viability* and *Preference* are controlled genetically, and are diploid and heritable following Mendelian rules of inheritance. Thus, each trait is determined by two alleles, with one allele inherited from each the mother and father. All alleles are explicitly represented in the model. Traits are expressed as the arithmetic mean of the two alleles carried, an assumption made in previously published models of sexual selection [35]. Individuals senesce as they *Age*. The trait, *Viability*, is a numeric variable that can confer a survival advantage, with larger values of the variable conferring better survival. The magnitude of this advantage is scaled by the global parameter *ViabilityEffect*. Males directly display their level of *Viability* through the variable *Signal*. However, the relationship between *Viability* and *Signal* may be altered by the global parameter *Disruption*.

The second heritable trait, *Preference*, is only expressed by females and determines a female’s ‘choosiness’ for a mate; females with higher levels of *Preference* will only mate with males with high *Signal*. Females have three further state variables: 1) *Pregnant*, i.e. “true” or “false”; 2) *Gestation*, i.e. the number of time steps for which they have been pregnant and 3) *PaternalAlleles*, four variables that represent the alleles of the male with which a female has mated.

To prevent population numbers from growing exponentially, density dependence acts on juveniles via the global variables *CarryingCapacity* and *JuvenileNumber*. The global variables *GestationLength*, *Fecundity*, *MaturityAge* and *Senescence* are life history parameters that determine for how long females are pregnant before giving birth, how many offspring a female gives birth to, the age at which individuals are able to reproduce, and longevity, respectively. We parameterised such aspects of the model to represent guppy life history.

All the parameters outlined here can be found in Table 1, and their mode of action is given below. A time step in the model corresponds to one day; simulations are usually run for several thousand-time steps. Space is not represented.

**Table 1:** Descriptions of model parameters, the values (*italicised* values are specific to guppy life history) and range over which they have been used in simulations.

Parameter	Relevant For	Description	Value
**Individual Variables**			
*Age*	All.	A count of the number of iterations since an individual was born.	Varies as the model iterates.
*Viability*	Adults, both sexes.	A numeric variable contributing to an individuals’ survival. The value is thearithmetic mean of two alleles (pseudo variables) carried.	Any positive value <1.
*Preference*	Adult females.	A numeric variable representing a female’s preference for mates with *Signal*.The value is the arithmetic mean of two alleles (pseudo-variables) carried.	Any positive value <1.
*Signal*	Adult males.	A numeric variable only carried by males, the value is chiefly determinedby *Viability* but also *Disruption*.	Equation 1.
*Pregnant*	Adult females.	A binary variable with the levels ‘true’ and ‘false’, indicating pregnancy.	True/False.
*Gestation*	Adult females.	A count of the number of iterations since a female became pregnant.	Varies as the model iterates.
*PaternalAlleles*	Adult females.	4 pseudo-variables, used to remember the allelic values of males withwhich the female has mated.	Allelic values of a mate.
**Global Variables**			
*GestationLength*	Adult females.	The number of iterations for which females are pregnant before giving birth.	*27*
*Fecundity*	Adult females.	The number of offspring that a female gives birth to.	*Drawn from a random-Poisson distribution, with lambda = 6.*
*Senescence*	Both	Scales the effect of *Age* on survival.	*100000*
*MaturityAge*	Both	The age at which individuals mature.	*60*
*JuvenileNumber*	Juveniles, both sexes.	A count of the number of juveniles currently alive in the environment.	Varies as the model iterates.
*CarryingCapacity*	Juveniles, both sexes.	Determines the population size by acting on juvenile survival.	2000
*ViabilityEffect*	Adults, both sexes.	The strength of the effect of *Viability* on survival.	0.3
*PreferenceMutation*	All.	The probability that a *Preference* allele will mutate at inheritance.	0.001 and 0.01.
*ViabilityMutation*	All.	The probability that a *Viability* allele will mutate at inheritance.	0.05.
*DisruptionDuration*	Adult males.	The number of iterations between time point A and B, during which *Signal* issubject to *Disruption*>0.	Varied at values of 500 through 5000 by 500.
*Disruption*	Adult males.	The magnitude of *Signal* disruption (Equation 1).	Varied at values of 0 through 0.7 by 0.1.

#### Process Overview and Scheduling

**Figure 1:**
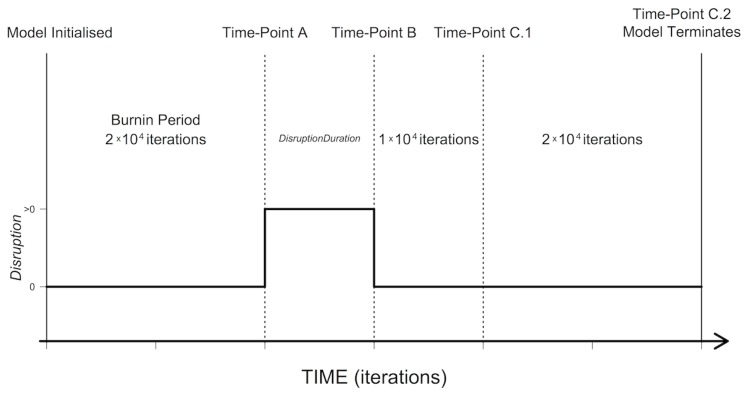
Overview of the Models Sampling Regime. A schematic of the periods and time-points in the model and whether environmental disruption (*Disruption*) of male-mating signals is present of absent at those time points. Mating signals are disrupted (i.e. *Disruption*>0) for the period *DisruptionDuration*. The actual length of *DisruptionDuration* and value of *Disruption* is varied between model runs (Table 1). At each time-point (A, B, C.1 and C.2) genetic data about individuals in the population are recorded.

At every time step, the following four processes (name of submodel given in brackets) are executed in the given order: 1) at certain time points (Fig. 1), an environmental perturbation of signal expression is introduced or removed (environmental disruption); 2) individuals either die or increase their *Age* (ageing and death); 3) females with *Pregnant* = ‘true’, give birth or increase *Gestation* (reproduction); 4) females with *Pregnant* = ‘false’, select a male and mate (mating). Individuals are processed in a randomized sequence and state variables are updated immediately after an individual performs an action.

#### Design Concepts

Individual behaviour is imposed via probabilistic rules. Females are able to sense the parameter *Signal* displayed by the males. Interaction occurs indirectly via density-dependent mortality of juveniles and directly via mating. Stochasticity is included in the model’s initialization to create initial genetic variation in all traits, and in all sub-models where probabilities of certain events are implemented via Bernoulli trials to represent demographic and genetic variability. Additionally, a random Poisson distribution determines fecundity at a given event.

To observe the evolutionary response of the simulated population, we observe the mean allelic values of all living individuals for: 1) *Preference* and 2) *Viability*. In both instances the paternally inherited allele was recorded, and preliminary model runs demonstrated that the same results are observed if the maternally inherited allele is used. These outputs were recorded at four time-points during a given model run (Fig. 1): 1) the time step immediately prior to the introduction of mating-signal disruption (time-point A); 2) the time step immediately prior to end of the disruption (time-point B); 3) 10,000 time steps after disruption has ceased (time-point C.1); and 4) 30,000 time steps after disruption has ceased (time-point C.2), immediately prior to the termination of a model run.

#### Initialization

The model is initialized with 75 males and 75 females. Each of these individuals is assigned an *age* from a random uniform distribution with a lower limit of zero and an upper limit of one thousand time steps (similar to a guppies maximum longevity). For mature females (i.e. *Age*>*MaturityAge*), *Pregnancy* is set ‘true’ with a probability of 0.5. Pregnant females are then assigned a *Gestation* from a random uniform distribution with a lower limit of one and an upper limit of *GestationLength*. For pregnant females, *PaternalAlleles* are assigned as randomly chosen floating point values between 0 and 1. Likewise, the alleles of all individuals are assigned random values between 0 and 1. These mechanisms initialise the model with genetic variability from which population levels of *Viability* and *Preference* evolve. Trait (phenotypic) values of *Viability* and *Preference* are then calculated as the arithmetic mean of the two alleles carried for that gene. Finally, males calculate their levels of *Signal* as via Eq. 1,
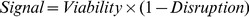
(1)where all parameters are as defined in Table 1. Thus, in the absence of an environmental disruption *Signal* directly conveys *Viability*.

#### Sub-models

Environmental Disruption: Environmental effects disrupt the expression of *Signal* via Eq 1.The stronger *Disruption,* the smaller the disparity between the *Signal* of males with differing *Viability*. At model initialisation, environmental effects are assumed to be absent and hence, *Disruption* is set to zero. The model is then given a burn-in period of 20,000 time steps (Fig. 1), simulating an established system of sexual selection that evolves from the genetic variation with which the model was initialised. Subsequently, a disrupting effect is introduced (time-point A, Fig. 1), and *Disruption* is set to an experimental level (Table 1) for a given period of time, *DisruptionDuration* (Fig. 1, Table 1). After this period, the disruption is removed from the environment and *Disruption* is set back to zero (time-point B, Fig. 1). Simulations then continue for 30,000 time steps. All the results presented here come from models that assume EDCs only affect the development of sexual signals in males, however such chemicals can also lead to sex-reversal [36], and likely have knock consequences for other aspects of fitness [37]. We tested the sensitivity of our conclusions to the presence of sex reversal with a short expansion of the model described above. That model simultaneously includes feminisation of males alongside reduction in sexual signal with EDC presence. The model incorporating feminisation produces qualitatively identical results to those described here in the main text. Larger differences between models incorporating feminisation and those not, may be expected under spatially explicit models as well as under differing assumptions about sperm limitation (Figs. S4, S5 and S6 and additional discussion in File S1: Supporting Information).

Aging and Death: During the aging and death submodel, each individual dies with a given probability. The probability of survival is calculated differently for juveniles and adults. For juveniles (i.e. *Age*<*MaturityAge*) survival is density-dependent, with the probability of surviving, being given by Eq. 2
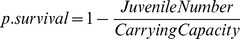
(2)where parameters are as given in Table 1. Thus, as the number of juveniles in the population approaches carrying capacity, juvenile probability of survival approaches 0. For adults, the probability of surviving is dependant on their *Age* as well as their level of *Viability* and the advantage conferred by *Viability*, given by Eq 3
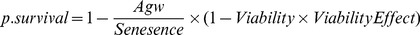
(3)where parameters are as given in Table 1. Thus as individuals age, they are more likely to die, but a higher level of *Viability* may confer a survival advantage depending on *ViabilityEffect*.

Reproduction: This sub-model applies to females with *Pregnant* = ‘true’. If *Gestation* equals *GestationLength*, a female gives birth to offspring, the number of which is determined by *Fecundity* (Table 1). Each offspring has an equal probability of being male and female. Each offspring is assigned, one allele from the mother and one allele from the father (stored in the females state variables; *PaternalAlleles*) for *Viability* and *Preference*. The alleles are randomly chosen from the two parental alleles with equal probability. Before an allele is assigned to an offspring, it may undergo mutation with a given probability; *PreferenceMutation* for *Preference* alleles and *ViabilityMutation* for *Viability* alleles (Table 1). If mutation occurs, the allele is assigned a random value between zero and one. Although guppies are diploid [38], we note that a number of groups of fish are largely polyploidy [39]; moving from a diploid to a polyploid system of inheritance in this model would have the same effect on the models outcome as increasing the number of alleles that contribute to the trait. Finally, the trait (phenotype) values *Viability* and *Preference* are calculated for all individuals (mean of allelic values). Offspring *Age* is set to zero. Male offspring set *Signal* according to Eq. 1, female offspring set *Pregnant* = ‘false’, *Gestation* = 0 and *PaternalAlleles* = 0. Here, we make the assumption that, whilst offspring a within a gravid female, they are sheltered from the effects of disruption on mating signals. Some data suggest that masculinising EDCs can affect offspring sexual development whilst in the mother, however such a response only seems to occur when the mother is directly fed large amounts EDC [40]. For lower background doses of EDCs, our assumption that the offspring is sheltered during pregnancy is probably sensible. Additionally, given that the majority of individuals will be exposed to disruption throughout life, the point at which that disruption has its effect will only alter the models output in a limited manner.

Mating: The mating sub-model only applies to females with *Pregnant* = ‘false’. Females randomly select a mate from all of the mature males with a level of *Signal* that is equal to or higher than her level of *Preference*. If no such male is available, the female does not mate on that iteration, thus imposing an opportunity cost to female selectivity. Where multiple males fulfil that criterion, each of those males has an equal probability of being selected as the mate. If a female does mate, she sets *Pregnant* = ‘true’, *Gestation* = 1, and the four alleles of the mated male are stored in the female’s state variables, *PaternalAlleles*.

### Model Parameter Values

Parameter values regarding life history (Table 1) were taken from the guppy literature [41]. To keep the degrees of freedom in parameter choice manageable, we identified regions in the parameter space representative of the kind of system we were interested in investigating, i.e. a mating system where females display preference for male mating signals.

Allelic values for *Viability* were bound at 0 and 1, thus allowing no (0) or some ‘theoretical maximum’ (1) survival advantage. To parameterize mutation rates and the *ViabilityEffect* parameter we explored the model without disruption to identify values that favoured the co-evolution of *Preference* and *Signal*, thus imitating sexual selection. We searched for combinations of parameter values that maximised the correlation between the resulting final population averages of *Preference* and *Signal*. Correlations were estimated based on the results of 200 model runs per parameter set using the *cor.test* function in *R* 3.0.1 [42]. Stronger correlations indicate settings conducive to the co-evolution of female preference for the males’ signals; i.e. strong inter-sexual selection. Of the values we tested, we found the strongest correlation between *preference* and *signal* was favoured when *ViabilityMutation* was high (0.05), *PreferenceMutation* was low (0.001) and *ViabilityEffect* was low (0.3) (Pearson’s product moment correlation coefficient (*r*) = 0.86). A complete set of said results and discussion thereof can be found in the supplementary material (Table S1 in File S1: Supporting Information). As observed, the difference between mutation rates of alleles of different genes, which maximizes the correlation between *Preference* and *Signal*, is extreme. Hence, models of disrupted signals were also run with a less extreme difference in mutation rates (*ViabilityMutation* = 0.05 and *PreferenceMutation* = 0.01), although the correlation between *Signal* and *Preference* was weaker (*r* = 0.72). In the following, we refer to the two genetic environments as those with low (0.001) and high (0.01) *PreferenceMutation*.

The probabilities of mutation in our model are high [43]. However, any model of sexual selection must maintain variance in the traits under sexual selection via some mechanism [44]. In reality, a suite of interacting factors may maintain variation in traits under sexual selection and exploring those effects is an active field of research [45]–[49]. Here, for simplicity, we assume a high level of mutation to implicitly represent all these processes, as has been done in previous models of sexual selection (e.g. Kokko et al. [44]).

To understand the effect of environmental disruption, we varied *Disruption* and *DisruptionDuration* (Table 1). The parameter space for these two variables was initially sampled sparsely. Once areas of parameter space that effectively captured a range of effects of *Disruption* and *DisruptionDuration* on the evolution of *Preference* had been identified, these areas were subject to more rigorous testing (i.e. sensitivity analysis). The values tested are given in Table 1 and each parameter set was tested with 100 model runs, giving a total of eight thousand model runs.

## Results

### Population Extinction

**Figure 2:**
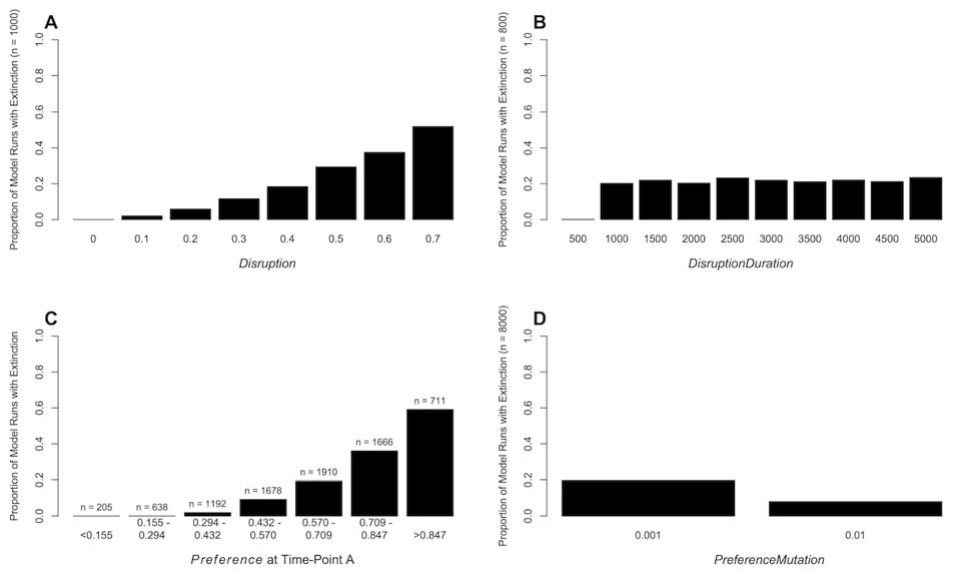
Factors Affecting Extinction After Disruption of Mating Signals. The proportion of model runs that resulted in population extinction with (A) varying *Disruption*, (B) varying *DisruptionDuration*, and (C) the level of *Preference* at time-point A (at the paternally inherited allele immediately prior to disruption of mating signals). In panels A, B and C the *PreferenceMutation* is 0.001, favouring a strong correlation between *Signal* and *Preference*. The presented data are from full model runs where *Disruption* and *DisruptionDuration* were co-varied (see section “Parameter Settings” for an overview of parameter values). Panel (D) represents the total proportion of experimental model runs resulting in population extinction with differing levels of *Preference.Mutation* (0.001 and 0.01). *Disruption and DisruptionDuration* were co-varied and other parameters were fixed at levels given in Table 1.

On a number of model runs, population extinction occurred following the disruption of mating signals. Extinctions occurred when females forwent mating because males displayed ‘unsatisfactory’ *Signal*. Under low *PreferenceMutation*, extinctions occurred on 19.6% of runs (*n* = 1568/8000) and visual exploration of the data suggested extinctions were predicted by three variables. Firstly, extinctions occurred relatively infrequently at weak *Disruption*, and became more frequent as *Disruption* became stronger (Fig. 2A). Secondly, *DisruptionDuration* had a threshold effect on extinction, with extinctions being observed frequently at values over one thousand (Fig. 2B). This threshold was determined by the generation time in the model (i.e. once males with high levels of *Signal* had died out), and hence would likely be species specific. Finally, *Preference* at time-point A predicted extinctions, with extinctions occurring more frequently at higher levels of *Preference* (Fig. 2C). Hence, extinctions were more common when females were choosy.

Under high *PreferenceMutation*, extinctions were predicted by the same three parameters as those with low *PreferenceMutation* (Fig. S1 in File S1: Supporting Information), but occurred on only 8.05% of model runs (n = 644/8000, Fig. 2D). These contrasting results highlight the importance of variation in *Preference* in preventing population extinction. Under such a scenario *Preference* was maintained by a higher mutation rate (*PreferenceMutation* = 0.01), which was a key factor in facilitating population persistence in the presence of *Disruption*. Higher mutation rates generated alleles for low *Preference*, which were necessary for females to adapt to the lower levels of *Signal* being expressed by males in the presence of *Disruption*.

### The Effects of Environmental Disruption on Preference and Viability

**Figure 3:**
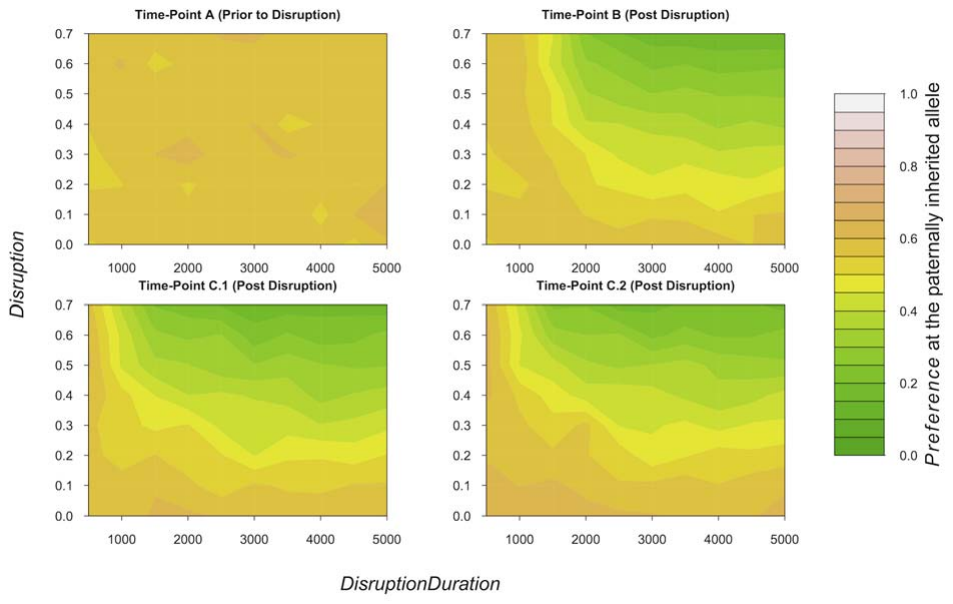
The Effects of Disruption on Mating Preference. The effects of co-varying *Disruption* and *DisruptionDuration* on the mean level of *Preference* at the paternally inherited allele of all living individuals at time-point A (immediately prior to signal disruption), time-point B (immediately after signal disruption has ended), time-point C.1 (ten thousand iterations after time-point B), and time-point C.2 (thirty thousand iterations after time-point B). If a model run resulted in extinction post disruption, data from time-points post extinction on that model run have been removed. Therefore, sample sizes vary for different time points. Time-point A *n* = 8000, time-point B *n* = 6679, time-point C.1 *n* = 6432, Time-point C.2 *n* = 6432. *PreferenceMutation* = 0.001 and all other parameters, except those on the x and y axes, were fixed at values given in Table 1.

We then explored the effects of co-varying *Disruption* and *DisruptionDuration* with low *PreferenceMutation,* as these settings maximised the correlation between *Signal* and *Preference*. At time-point A (prior to the mating signal disruption), the average level of *Preference* (at the paternally inherited allele) was just over 0.575, with some stochastic variation (Fig. 3). At time-point B (at the end of the disruption period), *Disruption* had reduced *Preference*. The extent to which *Preference* was reduced by *Disruption* and *DisruptionDuration* was proportional to the magnitude of these variables in an interactive manner (Fig. 3). When *Disruption* and *DisruptionDuration* were set at low values, *Preference* was only mildly affected. When *Disruption* and *DisruptionDuration* were both set at high values, *Preference* was reduced by a large amount (Fig. 3). However, if either *DisruptionDuration* or *Disruption* was low (500–1000 or ≤0.2, respectively), the other variable could be relatively high value, and only mild reductions of *Preference* occurred (Fig. 3). In the 10.000 and 20.000 iterations after disruption of mating signals (time-points C.1 and C.2), *Preference* typically remained low, except at very low levels of environmental disruption; e.g. at *Disruption* = 0.1 through 0.3 and *DisruptionDuration* = 500 through 2000 (Fig. 3).

**Figure 4:**
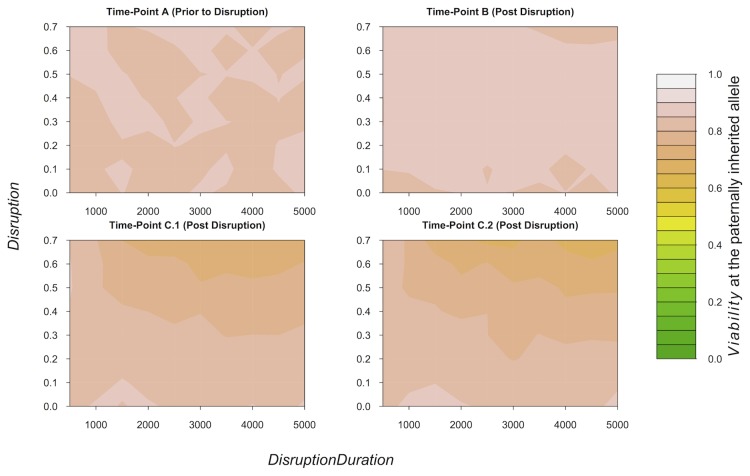
The Effects of Disruption on Viability. The effects of co-varying *Disruption* and *DisruptionDuration* on the mean level of *Viability* at the paternally inherited allele of all living individuals at time-point A (immediately prior to signal disruption), time-point B (immediately after signal disruption has ended), time-point C.1 (ten thousand iterations after time-point B), and time-point C.2 (thirty thousand iterations after time-point B). If a model run resulted in extinction post disruption, data from time-points post extinction on that model run have been removed. Therefore, sample sizes vary for different time-points. Time-point A *n* = 8000, time-point B *n* = 6679, time-point C.1 *n* = 6432, Time-point C.2 *n* = 6432. *PreferenceMutation* = 0.001 and all other parameters, except those on the x and y axes, were fixed at values given in Table 1.

At time-point A, *Viability* (at the paternally inherited allele) was high with minor variation (∼0.85–0.9; Fig. 4). At time-point B, *Viability* remained high but was slightly less variable than at time-point A (Fig. 4). At time-points C.1 and C.2, declines in *Viability* were associated with high levels of *Disruption* and *DisruptionDuration* (Fig. 4).

### Disruption of Mating Signals Under High and Low *PreferenceMutation*

Finally, we compared the effects of varying *Disruption* at high (0.01) and low (0.001) levels of *PreferenceMutation* with *DisruptionDuration* fixed at 5000. Complete sets of results from model runs with high *PreferenceMutation* can be found in the supporting material (Figs. S1, S2 and S3 in File S1: Supporting Information).

**Figure 5:**
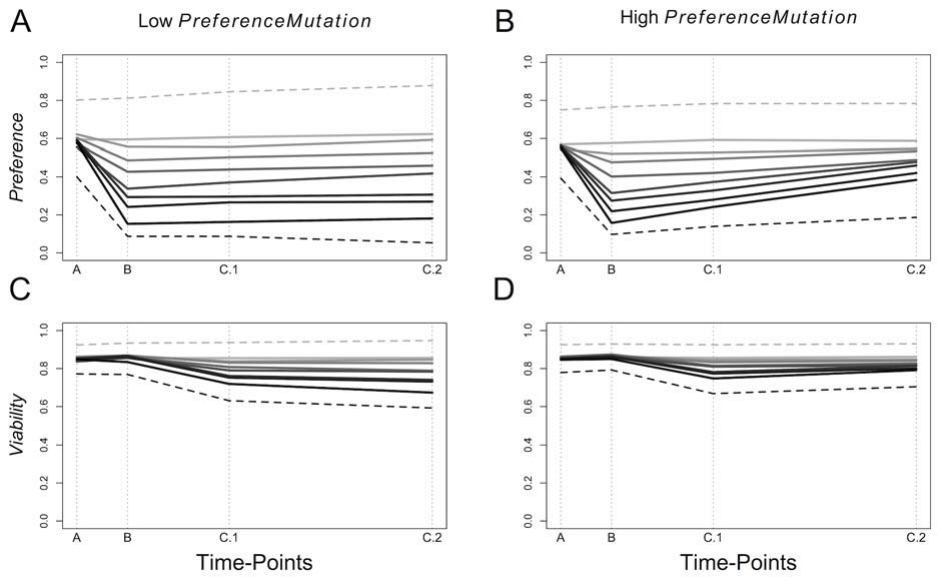
The Effects of Disruption Under Differing Levels of Allele Mutation. The effects of *Disruption* at different time-points (A, B, C.1 and C.2) under varying levels of *Disruption* (0 through 0.7 by 0.1 = grey scale, light to black) on A) *Preference* under low mutation for preference alleles (*MutationPreference* = 0.001), B) *Preference* under high mutation for preference alleles (*MutationPreference* = 0.01), C) *Viability* under low mutation for preference alleles (*MutationPreference* = 0.001) and D) *Viability* under high mutation for preference alleles (*MutationPreference* = 0.01). All estimates are based on the mean allelic values carried by all living individuals at their paternally inherited alleles. Dashed lines are means +1SD at *Disruption* = 0 (light grey) and means - 1SD at *Disruption* = 0.7 (black). All parameters were fixed at the values given in Table 1 and *DisruptionDuration* = 5000. If a model run resulted in extinction post disruption, data from time-points post extinction on that model run have been removed.

Slightly lower mean levels of *Preference* evolved at time-point A with high *PreferenceMutation* than with low *PreferenceMutation* (Figs. 5A and 5B). With both high and low *PreferenceMutation*, *Preference* was reduced at time-point B relative to time-point A, with greater reductions in *Preference* seen with higher levels of *Disruption*. As discussed above, *Preference* remained largely reduced at time-points C.1 and C.2 with low *PreferenceMutation* (Fig. 5A). However, with high *PreferenceMutation* promoting genetic variation in *Preference*, levels of *Preference* began to recover at time-points C.1 and C.2 (Fig. 5B).

The levels of *Viability* at time-point A were similar with high and low *PreferenceMutation* (Figs. 5C and 5D). In the periods after *DisruptionDuration*, *Viability* was reduced under both high and low *PreferenceMutation*, although to a greater extent in the latter and with increasing *Disruption* (Figs. 5C and 5D). Recovery of *Viability* was observed by time-point C.2 under high *PreferenceMutation,* but not under low *PreferenceMutation* (Figs. 5C and 5D).

## Discussion

The main aim of this study was to test to what degree and how fast female preference was lost in the presence of disruptions to the accuracy of male mating signals to convey information about male genetic quality. We also intended to establish insight into the capacity for the population to recover after disruption. We found that when the environment had a strong disruptive effect and female preference evolved to be highly stringent, the population could be driven to extinction (discussed in detail below). However, when the population was not driven to extinction, our results were in line with previous predictions [10]; we saw that environmental effects that disrupted the reliability of traits which signalled male quality reduced the population-average level of female preference for that signalling trait. We also saw reductions in mean viability of individuals within the population.

The level and duration of loss of preference was correlated with the strength of the disruption present. For most levels of disruption, after around two thousand iterations (about 24 simulated generations), persistent reductions in female preference for male mating signals were noticeable. Anthropogenic disruptions of mating signals have been recognised for over a decade [50], [51] and are likely to have been active for even longer. For example, it is around 20 years since the publication of initial studies describing the presence of EDC effects in fish [52], [53]. Hence, in areas where chronic EDC-contamination disrupts male mating signals, short-lived species may already display decreased mating preferences.

While we expected that ultimately the disrupting chemical would lead to a loss of female preferences, we were surprised by the effects on individual and population viability. Additionally, predictions about the effects of EDCs on individual viability were difficult to make, as it was unclear whether viability selection alone would maintain this trait. Initially in our model, sexual selection acted against mutations that reduced mean viability, because ‘choosy’ females would not mate with males carrying these mutations. However, after disruption of mating signals (time-points C.1 and C.2) female preference was reduced to such an extent that sexual selection against deleterious mutations relaxed and, ultimately, mean viability declined. Where preference re-evolved after disruption (i.e. with high *PreferenceMutation*), sexual selection promoted a recovery in population viability. Our observations agree with hypotheses that sexual selection via good-genes mechanisms can increase mean population-level viability, as mate-choice increases the frequency of highly viable individuals [54], [55]. What is more, this hypothesis is supported by recent experimental evidence [56], [57]. We do note, however, that this effect may not be universal; Arbuthnott and Rundle [58] failed to find similar effects in *Drosophila melanogaster*. Nevertheless, we suggest that for species in which sexual selection plays a role in purging deleterious alleles, disruption of mating signals may counteract this important effect.

We found that recovery of preference and viability after disruption was strongly associated with the rates of mutation of preference alleles. These results highlight the importance of understanding how additive genetic variation is generated and maintained in predicting how systems of sexual selection respond to rapid environmental changes. The importance of genetic variation for evolutionary responses to anthropogenic change has certainly not previously gone unnoticed in the literature [59], [60].

When females became highly selective and disruption of mating signals was strong, we observed population extinctions. Under such conditions, the signals displayed by males were shifted below the stringent level of preference that evolved. Therefore, females did not mate as males displayed ‘substandard’ signals; sometimes termed the ‘wallflower effect’ [61]. Jennions and Petrie [62] noted that where time constraints play a role, females may go unmated as a result of displaying mating preferences. However, such a scenario seems unlikely to apply to species like guppies that breed year-round [41]. What is more, models have also shown that when wallflower effects are present, extreme mate preferences should not evolve [63].

The extinction risk that we observe could, therefore, be considered unrealistic because the extreme mating preferences underlying extinction are unlikely to evolve. However, in some species, females may well have evolved high levels of discrimination in the absence of a wallflower effect before disruption of mating signals makes mating failure a possibility. This form of ‘evolutionary trap’ [64] may not be relevant to all species. However, for species in which females have become extremely choosy, rapid loss of mating signals could pose a risk to population viability.

So far, no consensus on the nature of the relationship between extinction risk and sexual selection has been reached [16], [57], [65]. Candolin and Heuschele [16] found that most evidence suggests sexual selection has either no, or mildly maladaptive effects, on a population’s initial ability to adapt to a changing environment. They conclude that sexual selection most likely negatively influences population persistence in the face of rapid environmental change [16]. The form of disruption that we fitted to male mating signals in our model would constitute a rapid change: within 6 to 12 generations, environmental disruption had reduced the level of male signals within the population to such an extent as the cost of female choosiness (a wallflower effect) increased extinction risk.

Our model contained a simplified mechanism of sexual selection. We felt that it would be best to begin with a simple model, as it allowed us to build a framework in which the results of the model remained tractable. Our approach may make our findings the result of ‘imposed’ or ‘rigid’ behaviours. For example, we use a strict model of mate choice, which underpinned the observed extinction. We have identified a number of aspects from which complexity may be added to the model to more realistically capture biological evolution. Table 2 outlines these areas as well as predictions of how these changes to the model may affect the evolutionary consequences of disrupted male mating signals.

**Table 2:** Future directions under which our model may be expanded to better capture the complexity of biological systems or to make further predictions about the effects of EDCs on systems of sexual selection.

Effect/Factor	Details	Model Predictions	Some Key References
**Mate Choice**	Female choice may be separated in to three ‘components’, 1) responsiveness,2) discrimination and 3) a preference function. We only varied responsiveness.Future models may address the other components, or allow females to vary onecomponent in relation to available males.	Flexible mate choice wouldincrease population resilienceto extinction resulting from awallflower effect.	[19], [61]
**Heritability**	We assume preference is heritable and genetically controlled. Studies havefound low heritability of preference in guppies, suggesting an environmentalcomponent in mate choice. In further models, past experiences or the suiteof available males may influence mate choice.	Environmental component tomate choice would decreasethe signal disruption associatedextinction the and loss ofpreference we report.	[19], [66]
**Preference Costs**	In our model, ‘opportunity cost’ of not mating is the only cost to preference.Other models have also imposed direct costs on female fitness; e.g. decreasedfecundity. Models of disrupted sexual selection may wish to include such costs.	Direct costs may result in lowerpreference levels. Weakerpreferences are less likely tobe susceptible to disruptedmating signals.	[44], [61], [63], [67]
**Good-Genes and/** **or Sexy-Sons**	In our model, breeding values of males result from improved offspring survivaland a ‘sexy-sons’ mechanism. Future work may model disruption of sexualselection operating purely via sexy sons. Wherein, breeding values associatedwith signalling males are purely the result of the procreation of ‘sexy’ sons.	Signal was maintained duringdisruption via its associationwith viability. Therefore, undera sexy-son’s-only mechanism,signal may be lost completely.	[44], [68]–[72]
**Genetic Control** **of Signal and** **Predation**	We assume that signals occur via an innate mechanism. In guppies, genes for colourare maintained by interacting sex linkage and predation. Models may also wish toinclude genetic suppression/promotion of signal and predation.	In the presence of disruptionand predation, alleles fornon-expression of colourmay proliferate becauseenvironmental disruptionsmarginalise the advantage ofcolour expression.	[59], [73]
**Multiple Sexually** **Selected Traits**	In many species, females select a male based on multiple traits; e.g. colorationand display rate in guppies. Our models contain a single trait. Further modelsof disrupted sexual selection may fit multiple traits under sexual selection.	One/multiple genes underlinkage may control preferencefor several traits. Hence,preference for a disrupted signalmay be maintained by selectionon preference for undisruptedtraits.	[12], [19], [21], [74]
**The Lek Paradox**	Here, high mutation rates counter the erosive effect of preference on geneticdiversity. Models may wish to explore the relative importance of other mechanismsthat maintain genetic variation with regard to disruption of mating signals. Forexample, multiple interacting loci may control preference/survival. Withadditional loci, the mutation rate at a single locus can be reduced, yet geneticvariation still maintained. Note the same outcome would be observedunder assumptions of polyploidy, rather than diploidy.	Unknown.	[43], [45], [46], [75]

As an example, consideration is given to processes operating guppies.

Nevertheless, our model showed that rapid environmental disruptions to the relationship between mating signals of male quality and underlying male fitness could alter the dynamics of female mate-choice and even mean population fitness. What is more, these effects can remain after the removal of the responsible environmental change. Short-term behavioural studies may be used to better parameterize models, allowing more precise long-term predictions to be made. We believe that ABMs represent an ideal system in which to further explore and generate predictions about the effects of disrupted male mating signals on systems of sexual selection. However, ultimately a suite of multi-generational experimental manipulations will be required to test the results presented here.

## Supporting Information

File S1 **Supporting Information.** Details of model testing, including Table S1, as well as details of our exploration of a model containing environmental sex reversal as a result of endocrine disrupting chemicals. Supplementary results (Figs. S1 through S6) are also given.
(DOCX)

File S2 **Model Code.** Model code for Netlogo and programming notes.
(RTF)
