# Supplementary material for: The Evolutionary Consequences of Disrupted Male Mating Signals: An Agent-Based Modelling Exploration of Endocrine Disrupting Chemicals in the Guppy
Source: PLoS One. 2014 Jul 21;9(7):e103100. doi: 10.1371/journal.pone.0103100 (PMC4105596; doi:10.1371/journal.pone.0103100)
Supplement: File S1 — Supporting Information. Details of model testing, including Table S1, as well as details of our exploration of a model containing environmental sex reversal as a result of endocrine disrupting chemicals. Supplementary results (Figs. S1 through S6) are also given. (DOCX) [file pone.0103100.s001.docx]

# The Evolutionary Consequences of Disrupted Male Mating Signals: an Agent-Based Modelling Exploration of Endocrine Disrupting Chemicals in the Guppy

# Supporting Information

Alistair McNair Senior^1,2,3^, Shinichi Nakagawa^1^ and Volker Grimm^4,5^

1. Zoology Department, The University of Otago, Dunedin, Otago, New Zealand.
2. The Charles Perkins Centre, The University of Sydney, Sydney, New South Wales, Australia.
3. School of Biological Sciences, The University of Sydney, Sydney, New South Wales, Australia.
4. Department of Ecological Modelling, Helmholtz Centre for Environmental Research, Leipzig, Saxony, Germany.
5. Institute for Biochemistry and Biology, University of Potsdam, Potsdam, Brandenburg, Germany.

*Corresponding author:

A. M. Senior

Email: alistair.senior1985@gmail.com

Tel: +64 (0) 3 479 5046

Fax: +64 (0) 3 479 7584

## Exploration of Mutation Rates and Viability Effects

As discussed in the main text (see section ‘Model Parameter Values’) we began by exploring the model in the absence of disruption of male mating signals to find those sets of *PreferenceMutaiton*, *ViabilityMutation* and *ViabilityEffect*, which maximised the correlation between the evolved level of *Viability* and *Signal*.

We tested all combinations of mutation rates *ViabilityMutation* (0.001, 0.01 and 0.05) and *PreferenceMutation* (0.001 and 0.01) and *ViabilityEffect* (0.3, 0.5 and 0.7). Each combination of parameters was run for 50,000 time steps and two hundred replicates. For the resulting final population averages of preference *Preference* and *Signal*, we searched for combinations or parameters that maximized the Pearson’s correlation between those values. Stronger correlations indicate a setting that is conducive to the co-evolution of female preference for the males’ signals; i.e. strong (inter-)sexual selection.

We found that strong correlations (sexual selection) tended to be favoured when the level of mutation at *Viability* was high and the level of mutation at *Preference* was low. Additionally, we found that a lower level of selection on *Viability*, favoured a higher correlation between *Preference* and *Signal* (Supplementary Table 1). Specifically, we found that, of the parameter values we tested, the correlation between *Preference* and *Viability* was maximized with a very high *ViabilityMutation* (0.05), a very low *PreferenceMutation* (0.001) and a low *ViabilityEffect* value (0.3); Pearson’s correlation coefficient between *Viability* and *Preference* was (*r*) = 0.86.

Under these settings, high *ViabilityMutation* and weak *ViabilityEffect* maintained variation in *Viability*, thus allowing choosy females to exercise their selective advantage over non-choosy females for a long period leading evolution of higher levels of preference. In contrast with high *ViabilityEffect* and/or low *ViabilityMutation*, genetic variability in *Viability* was eroded quickly and thus all females, choosy (high *Preference*) or non-choosy (low *Preference*) mated with similar high quality males.

## Sensitivity of the Model to Environmental Feminisation

#### Background

Exogenous chemicals such as endocrine disrupting chemicals (EDCs) can not only affect the expression of sexual signals, but may also affect development of phenotypic sex, potentially leading to sex reversal [1]. Here, we present a short expansion of the model described in the main text to test the sensitivity of our conclusions to the presence of sex reversal. This model incorporates feminisation of individuals alongside reduced expression of male sexual signals with increasing exposure to environmental chemicals.

#### Model

The model does not explicitly represent the genetic sex determination system of the species; in guppies this is a male heterogametic (or XY) system [2], similar to that in mammals. Previous models have shown that sex reversal can lead to a progressive loss of sex determining chromosomes [3,4]. However, those models also suggest that in systems of male heterogametic sex determination (i.e. guppies) feministation is unlikely to lead to the loss of a sex chromosome (unless at extremely high levels) and, in fact such systems return to a balanced genotypic sex ratio relatively quickly following the loss of feminising agents [4]. Thus, our choice not to explicitly model sex chromosomes seems reasonable in this instance. That being said, future models may wish to incorporate sex chromosomes, especially those focussing on feminisation in species of female heterogametic sex determination, where progressive loss of W chromosomes is a possibility.

Under the model described in the main text, the probability of developing as a male is fixed at 0.5. The model described here allows that probability to decrease, as the amount of environmental disruption increases, following Eq S1:

$p.male=\frac{e^{Disruption\times Feminisation}}{1+e^{Disruption\times Feminisation}}$, (Eq.S1)

where, *e* is the natural exponent, *Disruption* is the amount of chemical disruption in the environment (as described in the main text) and *Feminisation* is the feminising strength of the environmental chemical. All data presented here, fit a *Feminisation* slope of -3. Thus, when *Disruption* is equal to zero, the probability of developing as a male is 0.5, and as *Disruption* increases this value approaches zero with *Feminisation* dictating the strength of that slope; a detailed comparative analyses of such slopes can be found in [1].

The model incorporating the feminising effect was run under the same conditions as those described in the main text as high *PreferenceMutation*. Thus, the results presented here are comparable to those presented in Figs 2, 3 and 4 of the main text.

## Results and Discussion

The results produced by the model incorporating environmental feminisation, where qualitatively the same as those produced by the model without sex reversal. Extinction events were predicted by the same three factors as under the model not incorporating sex-reversal; 1) level of *Disruption*, 2) a threshold effect of *DisruptionDuration* and 3) *Preference* expressed by females at Time-Point A (Fig S4). We do note, that over all extinctions were perhaps slightly more prevalent in the presence of sex-reversal (comparison between Figs S4 and 2), although this may be expected given the fact that males become less prevalent, enhancing the ‘wall-flower’ effect described in the main text. With regards the effects of disrupted mating signals on *Preference* and *Viability*, again the results were similar both with and without sex reversal. Both traits were reduced by the presence of *Disruption*. The extent of loss of traits was predicted by *Disruption* and *DisruptionDuration* in an interactive manner (Figs S5 and S6).

The lack of effect of feminisation on our results can be traced to two factors. Firstly, the model described here is non-spatial. Thus, any allee effects [5], which may have resulted from a drop in the number of males within the population did not take hold. Secondly, we do not assume sperm limitation, thus a very small number of males are capable of fertilising all of the females within the population. Sperm is not thought to be limited under natural guppy populations [6], and if anything populations are thought to display a male biased operational sex ratio as a result of the number of females that are pregnant at any one time [7] (although we note that the absolute sex ratio of guppies can be female biased [8]). Although we did not observe any effect of sex reversal on our results here, future models especially those incorporating space explicitly, or those that make female preference a function of exposure to males, may report stronger effects of sex-reversal on the evolution of systems of sexual selection.

|  |  |  | *PreferenceMutation* = 0.001 |  |  | *PreferenceMutation* = 0.01 |  |
| --- | --- | --- | --- | --- | --- | --- | --- |
|  |  |  | *ViabilityMutation* |  |  | *ViabilityMutation* |  |
|  |  | 0.001 | 0.01 | 0.05 | 0.001 | 0.01 | 0.05 |
| *ViabilityEffect* | 0.3 | 0.197 | 0.549 | **0.858** | 0.145 | 0.376 | **0.723** |
|  | 0.5 | 0.130 | 0.324 | 0.820 | 0.157 | 0.352 | 0.711 |
|  | 0.7 | 0.196 | 0.273 | 0.569 | 0.053 | 0.116 | 0.516 |

## Table S1.

Correlations (*r*) between mean population level of *Preference* and *Viability* after fifty thousand iterations in the absence of mating signal disruption at varying levels of *ViabilityEffect*, *PreferenceMutation* and *ViabilityMutation.* All parameters are as described in Table 1 of the main text. Results are based on 200 models runs under each parameter set. Values in bold indicate the parameter settings under which the effects of disrupted mating signals were explored.

### Figure S1.

The proportion of model runs that resulted in population extinction with (A) varying *Disruption*, (B) varying *DisruptionDuration*, and (C) the level of *Preference* at time-Point A (immediately prior to disruption of mating signals). The presented data are from full model runs where *Disruption* and *DisruptionDuration* were co-varied (see section “Parameter Settings” for an overview of parameter values). *PreferenceMutation* = 0.01 and *Disruption* and *DisruptionDuration* were co-varied and other parameters were fixed at levels given in Table 1.

### Figure S2.

The effects of co-varying *Disruption* and *DisruptionDuration* on the mean level of *Preference* at the paternally inherited allele of all living individuals at time-point A (immediately prior to signal disruption), time-point B (immediately after signal disruption has ended), time-point C.1 (ten thousand iterations after time-point B), and time-point C.2 (thirty thousand iterations after time-point B). If a model run resulted in extinction post disruption, data from time-points post extinction on that model run have been removed. Therefore, sample sizes vary for different time points. Time-point A *n* = 8000, time-point B *n* = 7472, time-point C.1 *n* = 7356, Time-point C.2 *n* = 7356. *PreferenceMutation* = 0.01 and all other parameters, except those on the x and y axes, were fixed at values given in Table 1.

### Figure S3.

The effects of co-varying *Disruption* and *DisruptionDuration* on the mean level of *Viability* at the paternally inherited allele of all living individuals at time-point A (immediately prior to signal disruption), time-point B (immediately after signal disruption has ended), time-point C.1 (ten thousand iterations after time-point B), and time-point C.2 (thirty thousand iterations after time-point B). If a model run resulted in extinction post disruption, data from time-points post extinction on that model run have been removed. Therefore, sample sizes vary for different time-points. Time-point A *n* = 8000, time-point B *n* = 7472, time-point C.1 *n* = 7356, Time-point C.2 *n* = 7356. *PreferenceMutation* = 0.01 and all other parameters, except those on the x and y axes, were fixed at values given in Table 1.

### Figure S4.

The proportion of model runs that resulted in population extinction with (A) varying *Disruption*, (B) varying *DisruptionDuration*, and (C) the level of *Preference* at time-Point A (immediately prior to disruption of mating signals). The presented data are from full model runs where *Disruption* and *DisruptionDuration* were co-varied (see section “Parameter Settings” for an overview of parameter values). *PreferenceMutation* = 0.001 and *Disruption* and *DisruptionDuration* were co-varied and other parameters were fixed at levels given in Table 1. In addition to affecting the development of sexual signals, *Disruption* also resulted in feminisation.

### Figure S5.

The effects of co-varying *Disruption* and *DisruptionDuration* on the mean level of *Preference* at the paternally inherited allele of all living individuals at time-point A (immediately prior to signal disruption), time-point B (immediately after signal disruption has ended), time-point C.1 (ten thousand iterations after time-point B), and time-point C.2 (thirty thousand iterations after time-point B). If a model run resulted in extinction post disruption, data from time-points post extinction on that model run have been removed. Therefore, sample sizes vary for different time points. Time-point A *n* = 8000, time-point B *n* = 6526, time-point C.1 *n* = 6312, Time-point C.2 *n* = 6312. *PreferenceMutation* = 0.001 and all other parameters, except those on the x and y axes, were fixed at values given in Table 1. In addition to affecting the development of sexual signals, *Disruption* also resulted in feminisation.

### Figure S6.

The effects of co-varying *Disruption* and *DisruptionDuration* on the mean level of *Viability* at the paternally inherited allele of all living individuals at time-point A (immediately prior to signal disruption), time-point B (immediately after signal disruption has ended), time-point C.1 (ten thousand iterations after time-point B), and time-point C.2 (thirty thousand iterations after time-point B). If a model run resulted in extinction post disruption, data from time-points post extinction on that model run have been removed. Therefore, sample sizes vary for different time-points. Time-point A *n* = 8000, time-point B *n* = 6526, time-point C.1 *n* = 6312, Time-point C.2 *n* = 6312. *PreferenceMutation* = 0.001 and all other parameters, except those on the x and y axes, were fixed at values given in Table 1. In addition to affecting the development of sexual signals, *Disruption* also resulted in feminisation.

## References

1. Senior AM, Nakagawa S (2013) A comparative analysis of chemically induced sex reversal in teleosts: challenging conventional suppositions. Fish and Fisheries 14: 60-76.

2. Devlin RH, Nagahama Y (2002) Sex determination and sex differentiation in fish: an overview of genetic, physiological, and environmental influences. Aquaculture 208: 191-364.

3. Cotton S, Wedekind C (2009) Population consequences of environmental sex reversal. Conservation Biology 23: 196-206.

4. Hurley MA, Matthiessen P, Pickering AD (2004) A model for environmental sex reversal in fish. Journal of Theoretical Biology 227: 159-165.

5. Stephens PA, Sutherland WJ, Freckleton RP (1999) What Is the Allee Effect? Oikos 87: 185-190.

6. Pitcher TE, Rodd FH, Rowe L (2007) Sexual colouration and sperm traits in guppies. Journal of Fish Biology 70: 165-177.

7. Magurran AE (2005) Evolutionary Ecology: The Trinidadian Guppy; Harvey PH, May RM, editors. Oxford: Oxford University Press.

8. McKellar A, Turcotte M, Hendry A (2009) Environmental factors influencing adult sex ratio in Trinidadian guppies. Oecologia 159: 735-745.

## Figure S1

## Figure S2

## Figure S3

## Figure S4

## Figure S5

## Figure S6
