# Supplementary material for: The Evolutionary Consequences of Disrupted Male Mating Signals: An Agent-Based Modelling Exploration of Endocrine Disrupting Chemicals in the Guppy
Source: PLoS One. 2014 Jul 21;9(7):e103100. doi: 10.1371/journal.pone.0103100 (PMC4105596; doi:10.1371/journal.pone.0103100)
Supplement: File S2 — Model Code. Model code for Netlogo and programming notes. (RTF) [file pone.0103100.s002.rtf]

;;;;;;;;;;;;;;;;;;;;;;;;;;;;;;;;;;;;;:::::::::::::::;;; PARAMETERS ;;;;;;;;;;;;;;;;;;;;;;;;;;;;;;;;;;;;;;;;;;;;;;;;;;;;;;;;;;;;;;;;;;;;;;; Note that L (referred to as ViabilityEffect in the paper) scales the magnitude of the effect of f on survival and is programmed as a slider; Note that Zf and Zp (referred to as ViabilityMutation and PreferenceMutation in the paper) are programmed as sliders; Note that the variable Tab (referred to as DisruptionDuration in the paper) is programmed as an input box; G* (referred to as GestationLength in the paper) is the parameter specifying Gestation length i.e. how long a female is pregnant for; N (referred to as Fecundity in the paper) is ts number of offspring born by one female at a given time; X (referred to as Senescence in the paper) is the scaling aparmeter controlling adult mortality; A* (referred to as MaturityAge in the paper) is the age at maturity for guppies; C (referred to as CarryingCapacity in the paper)is the carrying capacity opporating on juvenile guppies; Y (referred to as Disruption in the paper), is the effect that disruption has on the relaitonship between Survival and Viability; Ystart is the value of Y at the begining of the model run - should usually be left at 0 to mimic an EDC free poulation, once; the period of disruption begins a variable called Yexp (programmed as a slider) becomes Y.; pop and var variables are recording variables, which remember population level data - they avoid runtime errors  globals [    G*  N     X  A*  C  Y    Ystart    pop.fm.A  pop.fp.A  pop.pm.A  pop.pp.A  var.fm.A  var.fp.A  var.pm.A  var.pp.A  pop.s.A  var.s.A  pop.p.A  var.p.A  pop.fm.B  pop.fp.B  pop.pm.B  pop.pp.B  var.fm.B  var.fp.B  var.pm.B  var.pp.B  pop.s.B  var.s.B  pop.p.B  var.p.B    pop.fm.C1  pop.fp.C1  pop.pm.C1  pop.pp.C1  var.fm.C1  var.fp.C1  var.pm.C1  var.pp.C1  pop.s.C1  var.s.C1  pop.p.C1  var.p.C1    pop.fm.C2  pop.fp.C2  pop.pm.C2  pop.pp.C2  var.fm.C2  var.fp.C2  var.pm.C2  var.pp.C2  pop.s.C2  var.s.C2  pop.p.C2  var.p.C2  ]; patches have no parameterspatches-own []; fm and fp are the maternal and paternal alleles carried for Viability, repectively; pm and pp are the maternal and paternal alleles carried for Preference for Signal, repectively; pfm pfp ppm ppp are psuedovariables (PaternalAlleles in the paper), which allow females to remember genetic informaiton about the partner which they mated with; f (Viability in the paper) is the gene for survival and is expressed by all individuals, it is the mean of fm and fp; p (Preference in the paper) is the gene for preference of signa l (s) and is expressed by females, it is the mean of pm and pp; s (Signal in the paper) is the signal of foraging ability expressed by males, it is dependant on f and Y (E); a (Age in the paper) is an individuals age; m (Pregnant in the paper) is a binary variable that specifies whether a female is pregnant or not; g (Gestation in the paper) is a variable specifying for how long a female has been pregnant; d (unmentioned but a progamming necessity) is a variable specifying the partner of a femaleturtles-own [      fm fp   pm pp  pfm pfp ppm ppp    f  p  s  a  m  g  d   ] ;;;;;;;;;;;;;;;;;;;;;;;;;;;;;;;;;;;;;;;;;;;;;;;;;;::;;; SET-UP ;;;;;;;;;;;;;;;;;;;;;;;;;;;;;;;;;;;;;;;;;;;;;;;;;;;;;;;;;;;;;;;;;;;;;;;;to setup       ;; (for this model to work with NetLogo's new plotting features,  ;; __clear-all-and-reset-ticks should be replaced with clear-all at  ;; the beginning of your setup procedure and reset-ticks at the end  ;; of the procedure.)  __clear-all-and-reset-ticks      ; below sets the global parameters      no-display    set G* 27  set N 6  set X 100000  set A* 60  set C 2000  set Ystart 0    set Y Ystart    set pop.fm.A 0   set pop.fp.A 0  set pop.pm.A 0  set pop.pp.A 0  set var.fm.A 0  set var.fp.A 0  set var.pm.A 0  set var.pp.A 0  set pop.s.A 0  set var.s.A 0  set pop.p.A 0  set var.p.A 0  set pop.fm.B 0   set pop.fp.B 0  set pop.pm.B 0  set pop.pp.B 0  set var.fm.B 0  set var.fp.B 0  set var.pm.B 0  set var.pp.B 0  set pop.s.B 0  set var.s.B 0  set pop.p.B 0  set var.p.B 0    set pop.fm.C1 0   set pop.fp.C1 0  set pop.pm.C1 0  set pop.pp.C1 0  set var.fm.C1 0  set var.fp.C1 0  set var.pm.C1 0  set var.pp.C1 0  set pop.s.C1 0  set var.s.C1 0  set pop.p.C1 0  set var.p.C1 0      set pop.fm.C2 0   set pop.fp.C2 0  set pop.pm.C2 0  set pop.pp.C2 0  set var.fm.C2 0  set var.fp.C2 0  set var.pm.C2 0  set var.pp.C2 0  set pop.s.C2 0  set var.s.C2 0  set pop.p.C2 0  set var.p.C2 0        ; creates a number of females (pink individuals)  ; sets their allelic values for p and f, then calculates the genetic (and phenotypic) values   ; finally gives them a random age and sets all other parameters to 0      crt 75   [         set color pink         set fm random-float 1     set fp random-float 1        set pm random-float 1     set pp random-float 1        set f (fm + fp) / 2    set p (pm + pp) / 2        set pfm 0     set pfp 0     set ppm 0     set ppp 0             set s 0    set a random-float 1000    set m "F"     set g 0    set d 0  ]        ; creates a number of males (blue individuals)  ; sets their allelic values for p and f, then calculates the genetic (and phenotypic) values   ; finally gives them a random age and sets all other parameters to 0 apart from s which is calculated      crt 75   [         set color blue         set fm random-float 1     set fp random-float 1        set pm random-float 1     set pp random-float 1        set f (fm + fp) / 2    set p 0        set pfm 0     set pfp 0     set ppm 0     set ppp 0         set s f * (1 - Y)    set a random-float 1000    set m "F"     set g 0    set d 0  ]            ; asks mature female turtles to become pregnant with a 50.50 chance  ; if you do become pregnant assigns random variables for gestation and offspring paternal genotypes          ask turtles with [color = pink and a >= A*][         ifelse random-float 1.0 > 0.5    [      set m "T"             set pfm random-float 1       set pfp random-float 1       set ppm random-float 1      set ppp random-float 1             set g random-float G*           ]    [set m "F"]      ]          reset-ticks    end;;;;;;;;;;;;;;;;;;;;;;;;;;;;;;;;;;;;;;;;;;;;;;;;;;;;;; GO ;;;;;;;;;;;;;;;;;;;;;;;;;;;;;;;;;;;;;;;;;;;;;;;;;;;;;;;;;;;;;;;;;;;;;;;;;;;  ; The go procedure, tells turtles what to do, it also controls whether EDCs are present using the first two lines of code  ; it also records data - this is only really important to prevent run time errors in behaviour spaceto go     if ticks = 20000 [set Y Yexp]   if ticks = 20000 + Tab [set Y Ystart]        ask turtles [senece]    ask turtles [reproduce]   ask turtles [mate]      record-data         tick       if ticks >= 20000 + Tab + 30000 + 1 [stop]        end;;;;;;;;;;;;;;;;;;;;;;;;;;;;;;;;;;;;;;;;;;;;;;;;; Submodels for the go procedure ;;;;;;;;;;;;;;;;;;;;;;;;;;;;;;;;;;;;;;;;;;;;;;;;;;;;;;;;;;;;;;;;;;;;;;;;;;;;;;;;;;;;;;;;;;;;;;;;;;;;;;;;;;;;;;;;; senece ;;;;;;;;;;;;;;;;;;;;;;;;;;;;;;;;;;;;;;;;;;;;;;;;;;;;;;;;;;;;;;;;;   ; if you are juvenile, die with probability of the population density or set age + 1; if you are a mature individual die with your specific probabiltiy or set age + 1to senece      ifelse (a <= A*)    [ifelse (random-float 1.0 > 1 - count turtles with [a <= A*] / C) [die] [set a (a + 1)]]   [ifelse (random-float 1.0 > 1 - (a / X) * (1 - f * l)) [die] [set a (a + 1)]]    end;;;;;;;;;;;;;;;;;;;;;;;;;;;;;;;;;;;;;;;;;;;;;;;;;;;;;;;;;;;;;;; reproduce ;;;;;;;;;;;;;;;;;;;;;;;;;;;;;;;;;;;;;;;;;;;;;;;;;;;;;;;;;;;;;;; only applies to pregnant females; if your gestation has reached the gestation age; then hatch N offspring; give all your offspring the charcteristics necessary e.g. age etc; also give them a sex and genotype following mendelian inheritance ; set their phenotypes; then set your pregnancy status back to F and also your pseudovariables for partners genetics 0; if you were not of a gestation equal to gestation age increase gestation by 1to reproduce      if m = "T" and color = pink     [    ifelse g >= G*     [             hatch random-poisson N             [                set m "F"         set a 0        set g 0                ifelse (random-float 1.0 >= 0.5)        [set color pink]        [set color blue]                 ifelse (random-float 1 > 0.5)         [set fm fm]        [set fm fp]                        if random-float 1 < Zf [set fm random-float 1]                        ifelse (random-float 1 > 0.5)         [set fp pfm]        [set fp pfp]                if random-float 1 < Zf [set fp random-float 1]                        ifelse (random-float 1 > 0.5)         [set pm pm]        [set pm pp]                if random-float 1 < Zp [set pm random-float 1]                ifelse (random-float 1 > 0.5)         [set pp ppm]        [set pp ppp]                if random-float 1 < Zp [set pp random-float 1]                                set f (fm + fp) / 2 ;+ random-normal 0 0.1        set p (pm + pp) / 2 ;+ random-normal 0 0.1                set pfm 0        set pfp 0        set ppm 0        set ppp 0                set d 0                ifelse (color = blue)         [set s f * (1 - Y) set p 0]         [set s 0]                        ]             set m "F"      set pfm 0      set pfp 0      set ppm 0      set ppp 0          ]         [set g (g + 1)]  ]      end ;;;;;;;;;;;;;;;;;;;;;;;;;;;;;;;;;;;;;;;;;;;;;;;;;;;;;;;;;;;;;;;;;;;;;; mate ;;;;;;;;;;;;;;;;;;;;;;;;;;;;;;;;;;;;;;;;;;;;;;;;;;;;;;;;;;;; only carry out this procedure if there are mature males around to mate with; if you are a mature if female, carry out mating; if you do carry out mating, begin by being assigning a mate, they must be male, mature and have a level of s, ; that is agreeable with your level of p; then remeber genetic onformation about that mate and become pregnant and set your level of g to 1.to mate        if color = pink and a >= A* and m = "F" [          if count turtles with [color = blue and a >= A* and s >= [p] of myself] > 1[            set d one-of turtles with [color = blue and a >= A* and s >= [p] of myself]             ;recordmate            set m "T"       set g 1       set pfm [fm] of d       set pfp [fp] of d       set ppm [pm] of d       set ppp [pp] of d             set d 0    ]  ]  end;;;;;;;;;;;;;;;;;;;;;;;;;;;;;;;;;;;;;;;;;;;;;;;;;   record-data   ;;;;;;;;;;;;;;;;;;;;;;;;;;;;; a function to record demographic and genetic data about the population ; this funciton is defensive programming against runtime errors associted with sampling in behaivour space; e.g. the mean and variance functions will fail if there are too fewer turtles (less than 2) - these functions ; avoid a runtime error which would arise if attmepts to calculate var are made with less that 2 turtlesto record-data    if ticks = 20000 and count turtles with [color = pink] > 2 and count turtles with [color = blue] > 2 [            set pop.fm.A mean [fm] of turtles    set pop.fp.A mean [fp] of turtles    set pop.pm.A mean [pm] of turtles    set pop.pp.A mean [pp] of turtles    set var.fm.A variance [fm] of turtles    set var.fp.A variance [fp] of turtles    set var.pm.A variance [pm] of turtles    set var.pp.A variance [pp] of turtles    set pop.s.A mean [s] of turtles with [color = blue]    set var.s.A variance [s] of turtles with [color = blue]    set pop.p.A mean [p] of turtles with [color = pink]    set var.p.A variance [p] of turtles with [color = pink]  ]          if ticks = 20000 + Tab and count turtles with [color = pink] > 2 and count turtles with [color = blue] > 2 [            set pop.fm.B mean [fm] of turtles    set pop.fp.B mean [fp] of turtles    set pop.pm.B mean [pm] of turtles    set pop.pp.B mean [pp] of turtles    set var.fm.B variance [fm] of turtles    set var.fp.B variance [fp] of turtles    set var.pm.B variance [pm] of turtles    set var.pp.B variance [pp] of turtles    set pop.s.B mean [s] of turtles with [color = blue]    set var.s.B variance [s] of turtles with [color = blue]    set pop.p.B mean [p] of turtles with [color = pink]    set var.p.B variance [p] of turtles with [color = pink]  ]          if ticks = 20000 + Tab + 10000 and count turtles with [color = pink] > 2 and count turtles with [color = blue] > 2 [            set pop.fm.C1 mean [fm] of turtles    set pop.fp.C1 mean [fp] of turtles    set pop.pm.C1 mean [pm] of turtles    set pop.pp.C1 mean [pp] of turtles    set var.fm.C1 variance [fm] of turtles    set var.fp.C1 variance [fp] of turtles    set var.pm.C1 variance [pm] of turtles    set var.pp.C1 variance [pp] of turtles    set pop.s.C1 mean [s] of turtles with [color = blue]    set var.s.C1 variance [s] of turtles with [color = blue]    set pop.p.C1 mean [p] of turtles with [color = pink]    set var.p.C1 variance [p] of turtles with [color = pink]  ]    if ticks = 20000 + Tab + 30000 and  count turtles with [color = pink] > 2 and count turtles with [color = blue] > 2 [            set pop.fm.C2 mean [fm] of turtles    set pop.fp.C2 mean [fp] of turtles    set pop.pm.C2 mean [pm] of turtles    set pop.pp.C2 mean [pp] of turtles    set var.fm.C2 variance [fm] of turtles    set var.fp.C2 variance [fp] of turtles    set var.pm.C2 variance [pm] of turtles    set var.pp.C2 variance [pp] of turtles    set pop.s.C2 mean [s] of turtles with [color = blue]    set var.s.C2 variance [s] of turtles with [color = blue]    set pop.p.C2 mean [p] of turtles with [color = pink]    set var.p.C2 variance [p] of turtles with [color = pink]  ]          end;;;;;;;;;;;;;;;;;;;;;;;;;;;;;;;;;;;;;;;;;;;;;;;;;;;;;;;;;;;;;;;;;;;;;;;;;;;;
